# BACH2 represses effector programmes to stabilize Treg-mediated immune homeostasis - a new target in tumor immunotherapy?

**DOI:** 10.1186/2051-1426-1-S1-O14

**Published:** 2013-11-07

**Authors:** Rahul Roychoudhuri, Kiyoshi Hirahara, Kambiz Mousavi, David Clever, Michael Bonelli, Christopher Klebanoff, Vittorio Sartorelli, Yuka Kanno, Luca Gattinoni, Ena Wang, Hui Liu, Franco Marincola, Igarashi Kazuhiko, John O’Shea, Nicholas P Restifo

**Affiliations:** 1 Center for Cancer Research, National Cancer Institute, Bethesda, MD, USA; 2 Molecular Immunology and Inflammation Branch, National Institute of Arthritis, Musculoskeletal and Skin Diseases, Bethesda, MD, USA; 3 Department of Transfusion Medicine, NIH Clinical Center, Bethesda, MD, USA; 4 Department of Biochemistry, Tohoku University, Sendai, Japan

## 

Through their functional diversification, distinct lineages of CD4+ T cells play key roles in either driving or constraining immune-mediated pathology and anti-tumor immune responses. Transcription factors are critical in the generation of cellular diversity, and negative regulators antagonistic to alternate fates often act in conjunction with positive regulators to stabilize lineage commitment. Genetic polymorphisms within a single locus encoding the transcription factor BACH2 are associated with numerous autoimmune and allergic diseases including asthma, Crohn's disease, coeliac disease, vitiligo, multiple sclerosis and type 1 diabetes. While these associations point to a shared mechanism underlying susceptibility to diverse immune-mediated diseases, a function for Bach2 in the maintenance of immune homeostasis had not been established. We have found that Bach2 plays a broad role in maintaining immune homeostasis, by stabilizing Treg-mediated immunoregulatory capacity while repressing the differentiation programmes of multiple effector lineages in CD4+ T cells. Bach2 was required for efficient formation of regulatory (Treg) cells and consequently for suppression of lethal inflammation in a that was Treg cell dependent. Assessment of the genome-wide function of Bach2, however, revealed that it represses genes associated with effector cell differentiation. Consequently, its absence during Tregpolarization resulted in inappropriate diversion to effector lineages. In addition, Bach2 constrained full effector differentiation within Th1, Th2 and Th17 cell lineages. These findings identify Bach2 as a key regulator of CD4+ T-cell differentiation that regulates the systemic balance between tolerance and immunity. These findings have implications for the design of novel therapies aimed at disrupting immune tolerance while promoting anti-tumor effector responses.

